# Mitochondrial DAMPs Increase Endothelial Permeability through Neutrophil Dependent and Independent Pathways

**DOI:** 10.1371/journal.pone.0059989

**Published:** 2013-03-20

**Authors:** Shiqin Sun, Tolga Sursal, Yasaman Adibnia, Cong Zhao, Yi Zheng, Haipeng Li, Leo E. Otterbein, Carl J. Hauser, Kiyoshi Itagaki

**Affiliations:** 1 Department of Surgery, Beth Israel Deaconess Medical Center/Harvard Medical School, Boston, Massachusetts, United States of America; 2 Department of Orthopedic Surgery, Beijing Army General Hospital, Beijing, China; University of Illinois College of Medicine, United States of America

## Abstract

Trauma and sepsis can cause acute lung injury (ALI) and Acute Respiratory Distress Syndrome (ARDS) in part by triggering neutrophil (PMN)-mediated increases in endothelial cell (EC) permeability. We had shown that mitochondrial (mt) damage-associated molecular patterns (DAMPs) appear in the blood after injury or shock and activate human PMN. So we now hypothesized that mitochondrial DAMPs (MTD) like mitochondrial DNA (mtDNA) and peptides might play a role in increased EC permeability during systemic inflammation and proceeded to evaluate the underlying mechanisms. MtDNA induced changes in EC permeability occurred in two phases: a brief, PMN-independent ‘spike’ in permeability was followed by a prolonged PMN-dependent increase in permeability. Fragmented mitochondria (MTD) caused PMN-independent increase in EC permeability that were abolished with protease treatment. Exposure to mtDNA caused PMN-EC adherence by activating expression of adherence molecule expression in both cell types. Cellular activation was manifested as an increase in PMN calcium flux and EC MAPK phosphorylation. Permeability and PMN adherence were attenuated by endosomal TLR inhibitors. EC lacked formyl peptide receptors but were nonetheless activated by mt-proteins, showing that non-formylated mt-protein DAMPs can activate EC. Mitochondrial DAMPs can be released into the circulation by many processes that cause cell injury and lead to pathologic endothelial permeability. We show here that mitochondria contain multiple DAMP motifs that can act on EC and/or PMN via multiple pathways. This can enhance PMN adherence to EC, activate PMN-EC interactions and subsequently increase systemic endothelial permeability. Mitochondrial DAMPs may be important therapeutic targets in conditions where inflammation pathologically increases endothelial permeability.

## Introduction

Cellular damage due to mechanical trauma, hemorrhage and sepsis can release mitochondrial (mt) damage motifs like mtDNA and formyl peptides. We previously showed that these molecules can trigger inflammation and organ injury (*in vivo*) by activating PMN [1], [2]. Thus mtDNA and formyl peptides clearly act as systemic alarmins or damage-associated molecular patterns (DAMPs) [3], [4] The mechanisms by which mt-DAMPs (MTD) act on other cells however, and the molecular events by which MTD induce the systemic inflammatory response syndrome (SIRS) and organ dysfunction are poorly understood. It is known though, that a major mechanism by which innate immunity causes organ dysfunction is instigation of PMN-mediated increases in endothelial cell (EC) permeability. Here for the first time we test the hypothesis that human mitochondrial ‘damage’ motifs (like mtDNA and mt-proteins) released following cell injury *in vivo* can trigger endothelial permeability changes observed in acute lung injury and acute respiratory distress syndrome (ARDS) (ALI/ARDS). We also set out to determine whether these effects occur via MTD interactions with EC, PMN or both. To study these processes under the most realistic and relevant possible conditions, we created studies that centered on the changes in endothelial monolayer permeability occurring ‘in real-time’ when human EC were exposed to fragmented whole human mitochondria (MTD) or to purified mtDNA in the presence or absence of human PMN. We then focused on the molecular biologic and cell signaling events that led to the alterations in EC permeability seen in the translational models we had created.

## Materials and Methods

### Compliance

The study was approved by the Institutional Review Board (IRB) of Beth Israel Deaconess Medical Center, Boston, MA USA. Written consent was obtained from blood donors. The tissue samples obtained for isolation of human mitochondria were portions of surgical pathology specimens that were not needed for diagnostic purposes. All samples were de-identified.

### Preparation of MTD and mtDNA

Human tissues such as the margins of liver resections, portions of spleens removed due to injury and bone marrow reamed from the femur during fracture fixations were obtained from operations performed at Beth Israel Deaconess Medical Center (BIDMC). Whole mitochondria (mt) were prepared using a mitochondrial isolation kit from Thermo Fisher Scientific (Rockford, IL). For certain experiments, mitochondrial debris (MTD) fractions were prepared by sonicating whole mitochondria (prepared from human liver) five times for 30 s as previously described [2]. Thus MTD contains both mitochondrial proteins and mitochondrial DNA.

Activity of MTD samples was standardized using a bio-assay based upon the ability of MTD to elicit a [Ca^2+^]_i_ response in Fura 2-loaded human PMN equal to that induced by 1 nM fMLP [5]. This method allowed us to use mitochondria from different tissue sources with minimal variation in activity. Concentrations are therefore expressed as dilution factors from the initial mitochondrial suspension. Preparations from different individuals still varied slightly in their ability to increase endothelial permeability and application, even at the same concentration based on PCR cycles or on PMN Ca^2+^ response, does not guarantee identical changes in permeability. This approach is by necessity very different from using commercial agents that are purified and fully characterized, but also reflects real biologic variability.

Protease treated whole mitochondria (MT) or MTD were prepared by treating samples with QIAGEN Protease (Qiagen) [6] at 56°C for 20 min. The protease was then inactivated by heating at 75°C for 20 min. Mitochondrial DNA (mtDNA) was prepared from human liver samples using the mtDNA Extractor (R) CT Kit from WAKO Chemical (Richmond, VA). Activity and purity of the mtDNA was evaluated by quantitative PCR using mitochondria specific primers [2]. MTD and mtDNA samples were evaluated and contained no detectable endotoxin.

### Endothelial Cell Cultures and Chemicals

EA.hy926 cells [7], derived from the fusion of HUVECs with the human lung carcinoma cell line A549, were obtained from Dr. Cora-Jean C. Edgell (University of North Carolina at Chapel Hill, NC). These “EA” cells were maintained in DMEM medium with 10% FBS and penicillin and streptomycin in a CO_2_ (5%) incubator at 37°C [8]. We chose to use EA cells for the bulk of the studies described in this manuscript since they maintain stable morphological, phenotypic and functional characteristics similar to human macro-vascular endothelial cells. Primary endothelial cells from different donors have limited life spans and different characteristics from batch to batch. To generalize studies done in EA cells critical findings were confirmed by using human pulmonary artery endothelial cells (HPAEC) or human lung microvascular endothelial cells (HMVEC). HPAEC and HMVEC were purchased from Lonza (Walkersville, MD) and maintained in EGM-2 and EGM-2MV medium, respectively. Chloroquine (an inhibitor of endosomal acidification) and ODN TTAGGG (an inhibitor of mtDNA binding to TLR9) were purchased from Invivogen (Carlsbad, CA). Other chemicals were purchased from Sigma (St. Louis, MI).

### Human PMN Preparations

PMN were isolated fresh from healthy volunteer donor blood samples. Detailed methods for PMN preparation can be found elsewhere [9]. Briefly, PMN were isolated from heparinized whole blood using a one-step centrifugation procedure in “PMN” isolation medium (Thermo Fisher Scientific). The neutrophil layer was collected, osmolarity was restored and cells were then washed and suspended in HEPES buffer. Red blood cells were lysed briefly to increase PMN purity.

### EC Permeability Measurements

Transendothelial electrical resistance (TER) was measured by seeding endothelial cells onto Cysteine/fibronectin-pretreated 8-well gold microelectrode chambers (8W10E+, 40 micro-electrodes per well) connected to an ECIS system (Applied BioPhysics). This system measures changes in the impedance of microelectrodes underlying the monolayer when an alternating current (AC) is applied. The tiny AC current (<1 µA) and voltage changes across the cells (a few millivolts) that are used to make the measurements have no detectable effect on the cells [8], [10], [11]. Confluence is determined by impedance coming to a plateau after overnight incubation (37°C, 5% CO_2_) as per the manufacturer’s protocol. These measurements provide a highly sensitive real-time biophysical assay indicating the state of cell shape and focal adhesion. As recommended by the manufacture, we assess capacitance changes at 64,000 Hz to evaluate monolayer permeability. As previously published, we normalize capacitance values for each well, dividing the observed value by the capacitance at plateau just prior to treatment [8], [11]. Values from each microelectrode are then pooled at discrete time points and plotted versus time as the mean ± SD. As permeability increases, impedance and resistance decrease whereas capacitance increases.

### Western Blot Analysis

Whole cell lysates were prepared using RIPA buffer (Thermo Fisher Scientific, Rockford, IL) following the manufacturer’s protocol. Primary antibodies against phospho-p38 (Thr180/Tyr182), phospho-p44/42 MAPK (Erk1/2), p38 MAPK, p44/42 MAPK (Erk1/2) antibodies were purchased from Cell Signaling Technology (Beverly, MA). HMGB1 antibody was purchased from abcam (Cambridge, MA). We used either β-actin (Santa Cruz Biotechnology, Santa Cruz, CA) or α-tubulin (Sigma, St. Louis, MI) as a house keeping protein. ECL (Thermo Fisher Scientific) was used to visualize proteins recognized by antibodies. Detailed procedures can be found elsewhere [9].

### RNA and cDNA Preparation and Quantitative PCR

RNA and cDNA were prepared using the RNesay mini kit (Qiagen, Valencia, CA) and the SuperScript VILO cDNA Synthesis kit (Life Technologies, Carlsbad, CA), respectively. 100–200 ng of cDNA was applied per qPCR reaction. Expression levels for E-selectin, ICAM-1, CD11b, CD18, L-selectin, HMGB1, and TLR9 were all determined by qPCR using SYBR-Green technology and STEP-ONE-Plus (Life Technologies, Carlsbad, CA) with specific primers purchased from OriGene (Rockville, MD). The expression levels for Sphingosine kinase 1 (Sphk1) were determined by TaqMan qPCR using validated specific primers and probe (Life Technologies, Carlsbad, CA). 200 ng of cDNA was loaded per reaction. All data were further normalized using GAPDH as the housekeeping gene. The levels of GAPDH expression remained stable across all the experimental conditions used in these studies, further demonstrating the internal stability of this housekeeping control under the experimental conditions used.

### Human PMN-Endothelial Cell Adhesion Assay

PMN were incubated for 30 minutes at 37°C in the dark in HEPES buffer with 1 mM Ca^2+^ and 3 µM Calcein-AM. Calcein-loaded PMN were set aside for use as a standard curve. PMN-EC adhesion assays were performed as previously described [8]. Near-confluent EC monolayers were stimulated with mtDNA (10–20 µg/mL) or medium for 6 hours. Labeled PMN (2.5×10^5^) were added for 1 hour after which the plates were inverted and centrifuged at 200×*g* for 5 min to remove non-adherent PMN. The calcein fluorescence of remaining PMN was then measured using a fluorescent plate reader (SpectaMax: Molecular Devices, Sunnyvale, CA) set at an excitation wavelength of 485 nm and an emission wavelength of 520 nm.

### Intracellular Calcium Measurements by Calcium Imaging

EC calcium mobilization was determined using a fluorescent calcium imaging system where cells are attached to glass coverslips. Cells were loaded with Fura-2 [8] and the intracellular calcium concentration ([Ca^2+^]_i_) was monitored with a Basic InCyt Im2 calcium imaging system (Intracellular Imaging Inc., Cincinnati, Ohio). 20–30 cells were typically selected for each experiment. The ratio of fluorescence at 340/380 nm was used to indicate [Ca^2+^]_i_.

### Imaging of mtDNA Localization in HPAEC by Confocal Microscopy

To visualize localization of mtDNA in HPAEC, human mtDNA was labeled with Alexa 488 using the Ulysis Nucleic Acid Labeling Kit (Life technologies, Grand Island, NY) following the manufacturer’s protocol [12]. Free Alexa 488 was removed by Micro Bio-Spin P-30 (Bio-Rad, Hercules, CA). HPAEC were stimulated with Alexa 488 labeled mtDNA and the red vital dye FM4-64 (Life Technologies) to visualize endocytosis. Cells were incubated for 4 hrs and a brief treatment with NucBlue (Life technologies) was used to label nuclei. Medium was then replaced with Live Cell Imaging Solution (Life technologies). Live cells were imaged at 37°C with 5%CO_2_ using a Zeiss LSM 510 Meta Live Cell Inverted Confocal System.

## Results

### MTD Directly Increase Endothelial Permeability

**Figure 1:**
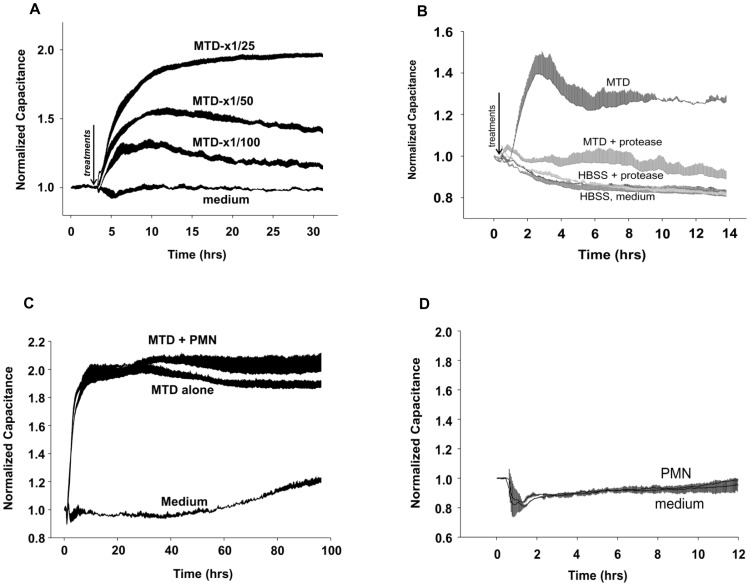
MTD-induced Permeability changes in EA and HPAEC. (A) EA cells were seeded onto ECIS cultureware (8W10E+). Various concentrations (×1/100, ×1/50, ×1/25) of MTD were applied to EA cells at time “0” and capacitance changes were determined as described previously [8]. (B) HPAEC were applied to ECIS cultureware as describe in 1A. The magnitude of responses is different from EA cells but MTD (×1/25) markedly increases permeability in HPAEC as well. Protease-treated MTD does not increase permeability. Proteases were always heat inactivated before application of MTD to HPAEC. MTD activity is unaffected by heat. Heat-treated protease itself (HBSS-protease), medium and HBSS have no effect on EC permeability. (C) MTD (×1/25), PMN (2×10^5^/well) from healthy volunteers were applied to EA cells along with MTD or in medium alone (N = 3 for MTD and MTD+PMN, N = 2 for Medium). (D) EA cells treated with medium or unstimulated PMN. These are control condition that apply to all ECIS experiments with EA cells. To avoid repetition, we show the typical medium or PMN (2×10^5^ cells/well) treated EA cell responses. For 1A, B, C: Values were collected every 160 sec at 64,000 Hz as recommended by manufacture. Data were collected from 2–3 wells and each well has 40 electrodes. Thus each data point represents mean and SD values from at least total of 80 electrodes. All experiments were repeated at least three times.

MTD applied to EA cells showed marked effects in endothelial permeability in the absence of PMN. As shown in **Figure 1A**, MTD dose-dependently increased endothelial permeability. To confirm the effects of MTD on primary human endothelial cells and show that its effect on permeability was not limited to one specific cell type, we applied MTD (×1/25) to human pulmonary artery endothelial cells (HPAEC). The changes seen were less than EA cells, but MTD still induced significant permeability increases (**Figure 1B**). Protease-treated MTD had no effect on permeability. Control conditions using heat-inactivated protease, medium and HBSS showed no effects on permeability. The addition of human PMN to EC monolayers already treated with MTD yields no additional effect on permeability (**Figure 1C**) and unstimulated alone PMN had no measurable effect on EC monolayer permeability (**Figure 1D**).

### MtDNA Increases EC Permeability both Directly and Indirectly via PMN

**Figure 2:**
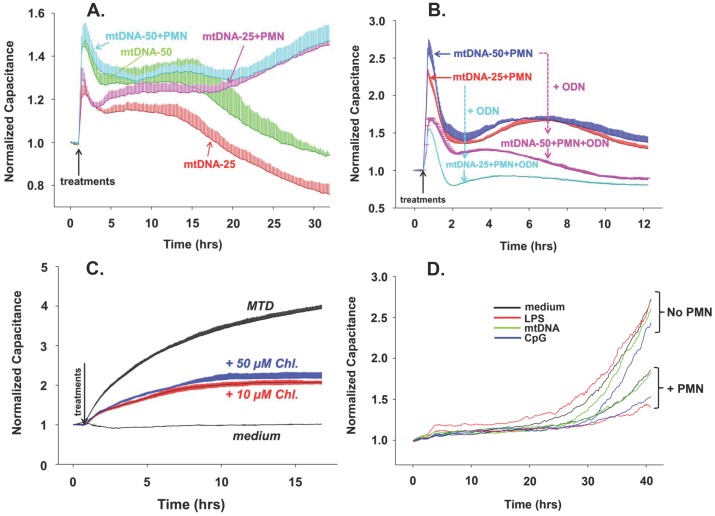
mtDNA and MTD induce permeability in EA calls. (A) mtDNA-induced endothelial permeability changes were evaluated by ECIS as described in Figure 1. EA cells were seeded onto ECIS cultureware and 25 or 50 µg/mL mtDNA was applied with or without 2–3×10^5^ human PMN. (B) In addition to the conditions in 2A, 5 µM ODN TTAGGG (a specific inhibitor of TLR9) was applied to chambers where indicated. Two or three wells were used per reaction, mean and SD values are shown. (C) EA cells were pre-treated with 10 or 50 µM Chloroquine then MTD (×1/25) were applied. Two or three wells are used per reaction, mean and SD values are shown. For 2A, B and C the conditions were the same as described in Figure 1. All experiments were repeated at least three times. (D) Unlike EA or HPAEC, HMVEC have very slow and limited responses to stimulation.

MtDNA (25 and 50 µg/mL) was prepared from mitochondria isolated from human liver and applied to EA cells with and without human PMN present. We note an immediate, dose-dependent rise in monolayer permeability both in the presence and absence of PMN (**Figure 2A**). In the absence of PMN however, permeability changes were transient and decayed relatively quickly. In the presence of PMN the increases in permeability were sustained and even intensified over time. We repeated this study in the presence of the oligodeoxynuclotide (ODN) TTAGGG, which is a selective inhibitor of endosomal nucleic acid receptors. [13] TTAGGG markedly attenuated the induction of permeability by mtDNA in the presence of human PMN (**Figure 2B**).

### Chloroquine Inhibits MTD-induced Permeability Changes

Hypomethylated DNA species like mtDNA are thought to be sensed through endosomal DNA receptors such as TLR 3, 7, 8 and 9 that require endosomal acidification. Since MT contain hypomethylated CpG DNA we examined the effects of chloroquine (CQ, an inhibitor of endosomal acidification [14]) on MTD-induced EC permeability in the absence of PMN. Endothelial cells pre-treated with CQ showed significant attenuation of MTD-induced permeability. This suggests that EC sensing of mtDNA via endosomal nucleic acid receptors contributes to the permeability increase caused by MT (**Figure 2C**).

### MtDNA Had Limited Effects on HMVEC

The effects of mtDNA or CpG with or without PMN on permeability of human pulmonary microvascular endothelial cells (HMVEC) with or without PMN were also evaluated. As seen in **Figure 2D**, the responses of microvascular EC to both endosomal and cell membrane TLR agonists were found to be limited and slow in these systems compared with the responses of EA cells or pulmonary artery EC.

### MtDNA is Taken Up into Endosomes by HPAEC

**Figure 3:**
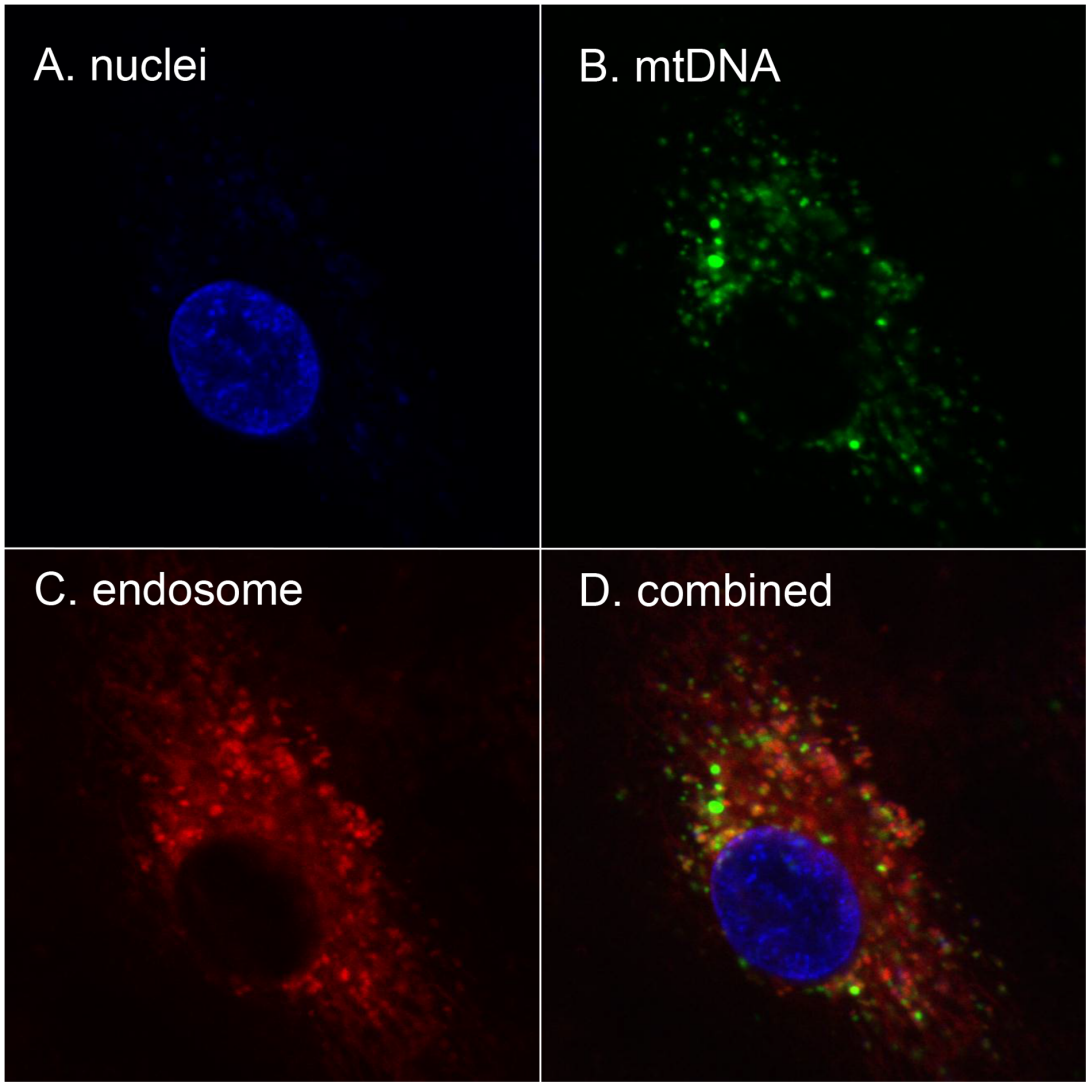
Externally applied mtDNA localizes to endosomes. HPAEC were reacted with mtDNA labeled with Alexa-488, FM4-64 (a marker for endosomes) and NucBlue (a marker for nuclei). After 4 hr of incubation, cells were examined for dye localization by confocal microscopy. A. nuclei, B. endosomes, and C. mtDNA were separately detected. Co-localization of mtDNA (Alexa 488) and the endosomal marker (FM4-64) is shown in D. Experiments were repeated at least three times.

DNA-sensing TLRs are endosomal in location. We therefore next sought to show that mtDNA applied to EC externally was taken up into endosomes. To do this, we applied mtDNA prepared from human liver and freshly labeled with Alexa 488 to HPAEC along with FM4-64, a marker that localizes to endosomes. We then examined live cells under confocal microscopy. As shown in **Figure 3**, mtDNA was noted to be taken up rapidly by HPAEC and to co-localize to the endosomes as determined using FM4-64.

### Mitochondrial Proteins Induce EC Permeability and Ca^2+^ Mobilization Directly

**Figure 4:**
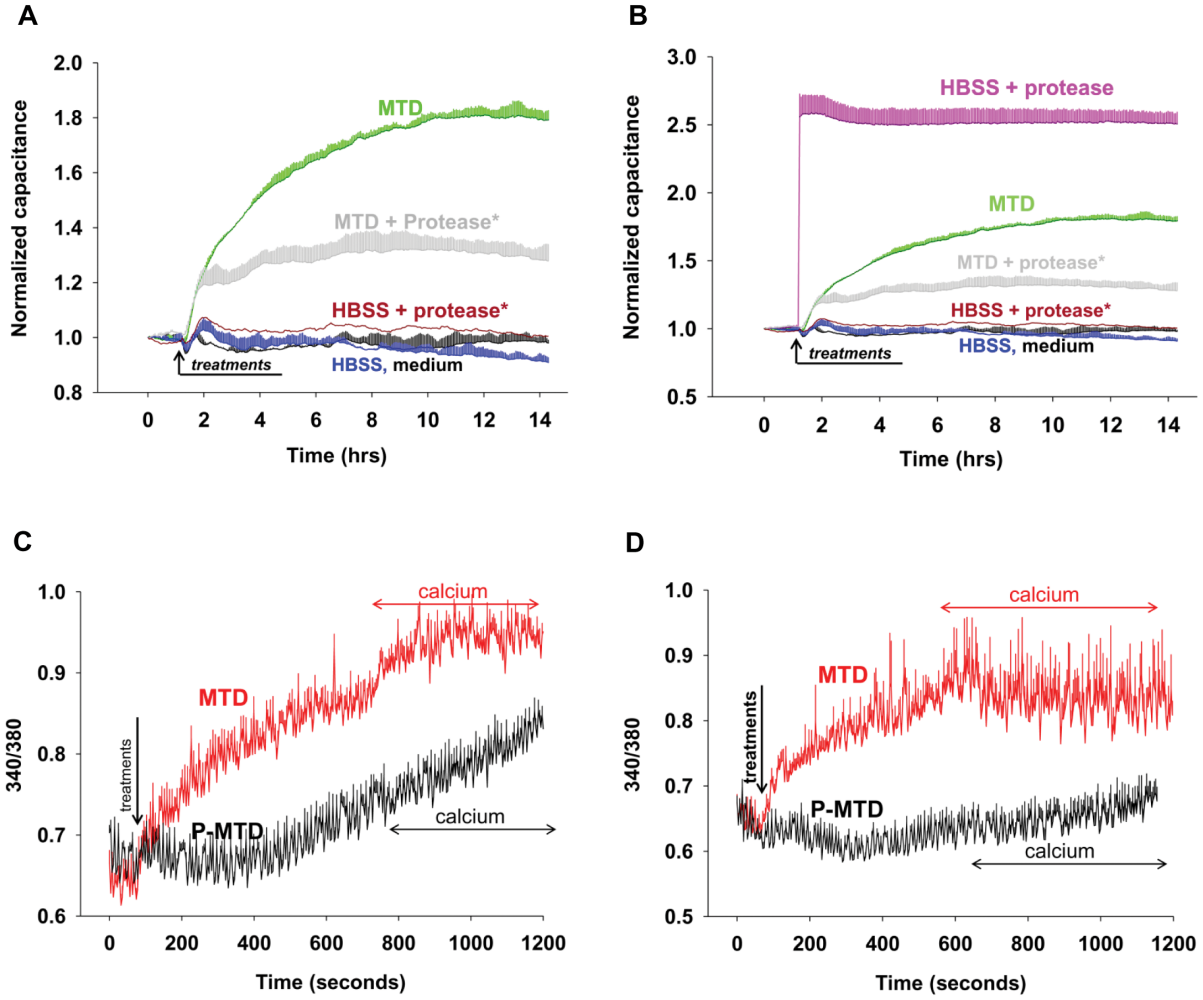
Protease-treatment of MTD reduces permeability increase and calcium mobilization. (A) MT with or without sonication (MTD, MT) were pretreated with protease. After heat inactivation of the protease, MT and MTD were applied to ECIS chambers as described before. Traces represent mean and SD values from at least two wells. Experiments were repeated at least three times. (B) In addition to Figure 4 data, HBSS with active protease was applied to EA cells. Active proteases instantly increase EC permeability. Experiments were repeated at least three times. 4C) and 4D): MTD with or without protease treatment (P-MTD, MTD) were applied to EA (4C) or HPAEC (4D) in the absence of extracellular calcium. Then 1.8 mM calcium was applied at the times indicated. The number of cells used for each measurement is shown in figures. Mean and SE values are shown. Experiments were repeated at least three times.

In addition to mtDNA, mitochondria contain proteins that EC might sense as alarmins. These could be formylated proteins synthesized in the mitochondria that act on formyl peptide receptors (FPR) like those on PMN, or they could be non-formylated proteins sensed via other mechanisms. We examined the permeability of EC exposed to MTD pre-treated with a protease cocktail or media only [6] **.** Protease activity was terminated by heat (75°C/20 min) prior to treating EC. As shown in **Figure 4A**, protease treatment of MTD markedly reduced their ability to induce an increase in EC permeability, although that reduction was incomplete. Heat treatment alone had no effect on the activity of MT. Also, heat-treated proteases (HBSS+protease) have no effect on EC permeability even though direct treatment of endothelial cells with active (i.e. not heat-treated) proteases dramatically increases permeability (**Figure 4B**).

Agonists like thrombin and histamine increase EC permeability in part by increasing cytosolic calcium concentrations ([Ca^2+^]_i_). We therefore applied MTD to EA cells or HPAEC to evaluate calcium mobilization (**Figure 4C and D**). Experiments performed in the absence of extracellular calcium showed that MTD (×1/25, applied at t = 60 sec) induced release of intracellular calcium stores in both EA cells and HPAEC. Addition of extracellular calcium (1.8 mM, applied 600–700 sec after MTD) showed that MTD also causes a Ca^2+^ influx. This was more prominent in the EA cells. Lastly, when MTD were pre-treated with a protease cocktail (P-MTD) the [Ca^2+^]_i_ increases were very limited.

### Formyl Peptides Do Not Induce EC Permeability Directly

**Figure 5:**
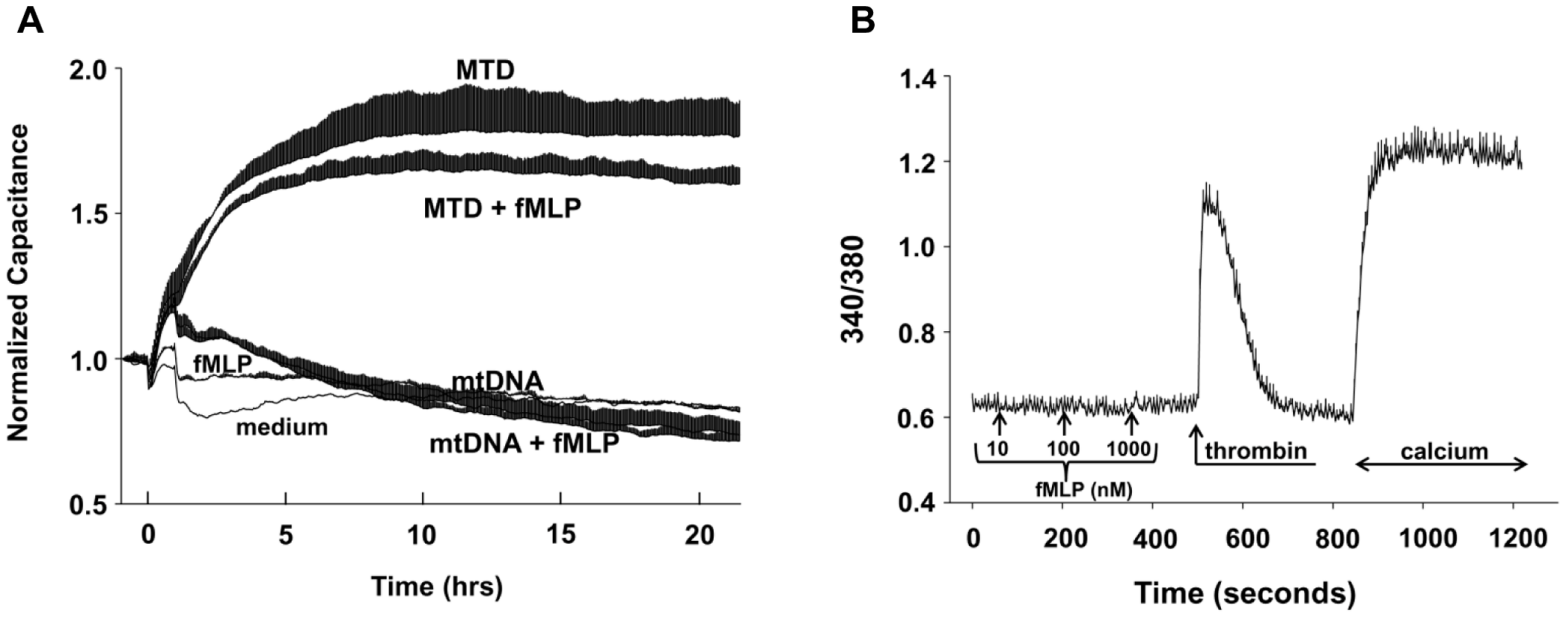
Formyl peptides have no effect on EC permeability or Ca^2+^ mobilization. (A) 10 nM fMLP was applied to EA cells in ECIS cultureware either separately or together with MTD or mtDNA. fMLP alone did not increase permeability and no additional increase was detected when it was applied with MTD. The number of wells per condition is shown in parentheses: medium (1), fMLP (2), MTD (3), MTD+fMLP (3), mtDNA (3), mtDNA+fMLP (3). Mean and SD values are shown. Each well contains 40 electrodes, thus each value comes from at least 40 individual values. Experiments were repeated at least three times. (B) Various concentrations of fMLP were applied and failed to induce any calcium depletion from the endoplasmic reticulum in EA cells. As a positive control, cells reacted readily to thrombin (1 U/mL), both releasing calcium from the ER and showing calcium influx (SOCE) after addition of 1.8 mM extracellular calcium. Trace represents mean and SD values from at least 20 cells. Experiments were repeated at least three times.

We next examined whether mitochondrial formyl peptides contribute directly or in an additive manner to the permeability of EC exposed to MTD or mtDNA. We found that fMLP (10 nM) had no direct effect on EC permeability. Moreover, application of fMLP to EC in addition to MTD or mtDNA had no additional effect on the permeability changes seen (**Figure 5A**). EA cells studied by fluorescence microscopy showed no [Ca^2+^]_i_ transients in response to fMLP despite responding briskly to thrombin stimulation. This suggests the absence of formyl peptide receptors (FPRs) (**Figure 5B**). Taken together, these data suggest that non-formylated MT proteins (of genomic origin) cause changes in EC permeability directly, and that exposure to non-formylated MT-proteins may play a significant role in the regulation of EC permeability after injury.

### MtDNA Activates PMN Adherence to EC

**Figure 6:**
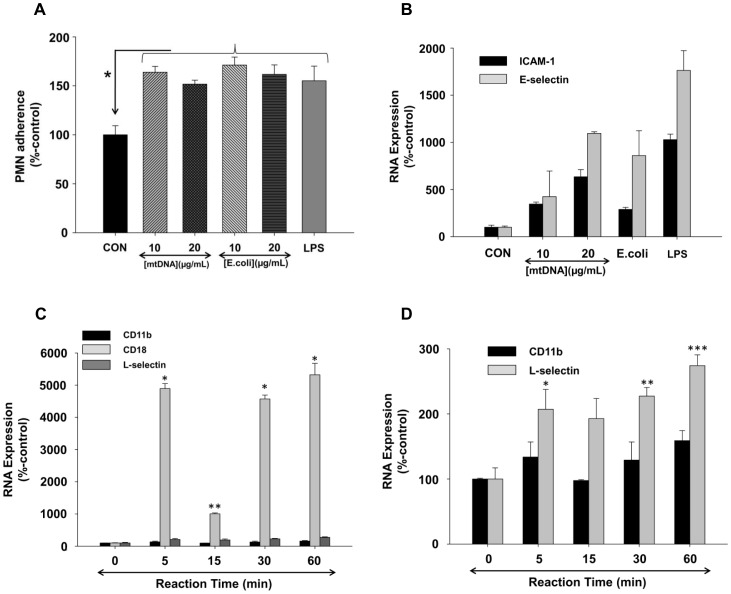
mtDNA-treatment increases PMN adherence to endothelium by increasing adherence molecules on both EC and PMN. (A) EA cells were treated by 10 or 20 µg/mL of mtDNA, E.coli DNA, or 10 ng/mL of LPS for 6 hrs. Calcein-loaded PMN were applied to EA cells for the last 1 hr of incubation and the percentage of PMN attached to the EA cells was determined as described in Methods. Data were normalized using medium-treated cells as a control (100%). Mean and SE values from at least 6 experiments are shown. Compared to the medium control, mtDNA, E.coli DNA, and LPS treatments all significantly increased PMN adherence to the EC. Experiments were repeated at least three times and studied by One-way ANOVA. (B) EA cells were incubated with various concentrations of mtDNA (10 and 20 µg/mL), E.coli DNA (10 and 20 µg/mL), and 100 ng/mL LPS for 6 hrs. 100 ng of cDNAs were prepared and loading was standardized. qPCR was then performed with specific primers for ICAM-1, E-selectin, and GAPDH. Data were normalized by GAPDH and medium-treated cells as control (100%). Mean and SE values are shown from at least n = 3. **6C) and 6D)** PMN (3 million cells) were stimulated with mtDNA (20 µg/mL) for the indicated times. cDNAs were prepared and qPCR was performed using CD11b, CD18, L-selectin and GAPDH primers. Data were normalized to GAPDH using medium-treated cells as the control ( = 100%). Mean and SE values from n = 6 are shown. (C): *: <0.001, **: = 0.036, (D): *: = 0.022, **: = 0.005, ***:<0.001, compared to time “0” value (analyzed by One Way ANOVA, SigmaPlot 11).

Next, we examined whether enhanced PMN-EC adherence might contribute to the increased permeability observed when PMN and EC were exposed to mitochondrial alarmins. EA cells were treated with mtDNA (10, 20 µg/mL) for 6 hrs and calcein-loaded PMN were applied to the EA cell monolayers for the last 60 min. Exposure to mtDNA significantly increased PMN adherence to EA cells (**Figure 6A**). We therefore treated EA cells with mtDNA and evaluated their expression of ICAM-1 and E-selectin by qPCR. We found expression of these adhesion molecules to be dramatically increased (**Figure 6B**). We also examined whether mtDNA increased PMN expression of the relevant counter-ligands and found that it markedly increased PMN expression of CD18b and L-selectin (**Figure 6** **C&D**). Thus exposure to mtDNA increases expression of selectins and integrins in both PMN and EC and causes them to adhere to each other.

### MtDNA Activates PMN Store Operated Calcium Entry (SOCE)

**Figure 7:**
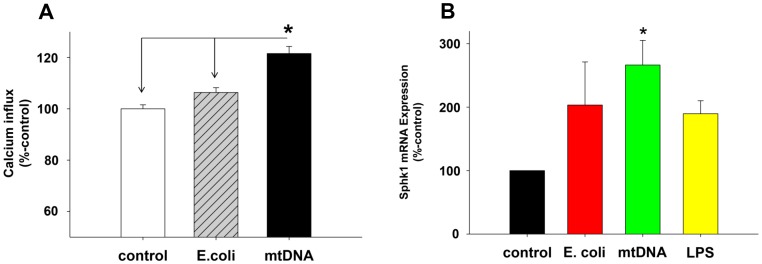
mtDNA increases SOCE and expression of Sphk1 in PMN. (A) Human PMN were treated with 20 µg/mL of E.coli DNA or mtDNA for 60 min then loaded with fura-2. Thapsigargin (1 µM) was applied to under nominally calcium-free conditions and then 1.8 mM extracellular calcium was applied at the indicated times. Calcium influx was calculated as described elsewhere [9] as the area under the curve for [Ca^2+^]_i_ (AUC) over 120 sec. Data were analyzed using medium-treated PMN as 100%. Mean and SE values are shown. At least 3 experiment were done per condition. * denotes a significant difference by student t-test (SigmaPlot 11) compared to time “0” value. Experiments were repeated at least three times. **(7B)** Freshly isolated human PMN (5 million cells in 2 mL) were incubated with medium, mtDNA (10 µg/mL), E.coli DNA (10 µg/mL), or LPS (100 ng/mL) for 60 min. Then RNA and cDNA were prepared using the RNesay mini kit (Qiagen) and SuperScript VILO cDNA Synthesis kit (Life technologies), respectively. 200 ng of cDNA per reaction was used for TaqMan qPCR assay for Sphk1 and GAPDH to evaluate expression levels. Sphk1 expression levels were then further normalized by GAPDH using the medium control result as 100%. Four different PMN preparations were used for stimulation and qPCR assays were done in triplicates. Mean and SE values from four different experiments are shown. Data were analyzed by One Way ANOVA. * denotes a significant difference between mtDNA treatment and the medium control (p<0.05). No other significant differences were found.

We previously showed that PMN are activated via increases in SOCE after clinical injury [15] although the mechanisms of that SOCE activation were unknown at the time. We now hypothesized that exposure to mitochondrial DAMPs might contribute to the increased PMN SOCE we found after clinical injury. We found that exposure to mtDNA did indeed increase thapsigargin (TG) induced PMN calcium influx significantly (**Figure 7A**). We have also previously shown [16] that enhanced SOCE in PMN was related to the increased generation of sphingosine 1-phosphate (S1P). Sphingosine kinase-1 (Sphk1) is the major bio-synthetic pathway for S1P synthesis. We therefore questioned whether exposure to mtDNA might increase PMN expression of Sphk1, thus increasing intracellular production of S1P and leading to increased [Ca^2+^]_i_. We observed (**Figure 7B**) that mtDNA treatment did indeed enhance PMN expression of Sphk1 mRNA. The data support a general hypothesis that release of mitochondrial DAMPs by injury can activate PMN-EC interactions by increasing PMN SOCE and thus activating [Ca^2+^]_i_ dependent PMN inflammatory responses.

### MTD Activate Endothelial Cell MAP Kinases

**Figure 8:**
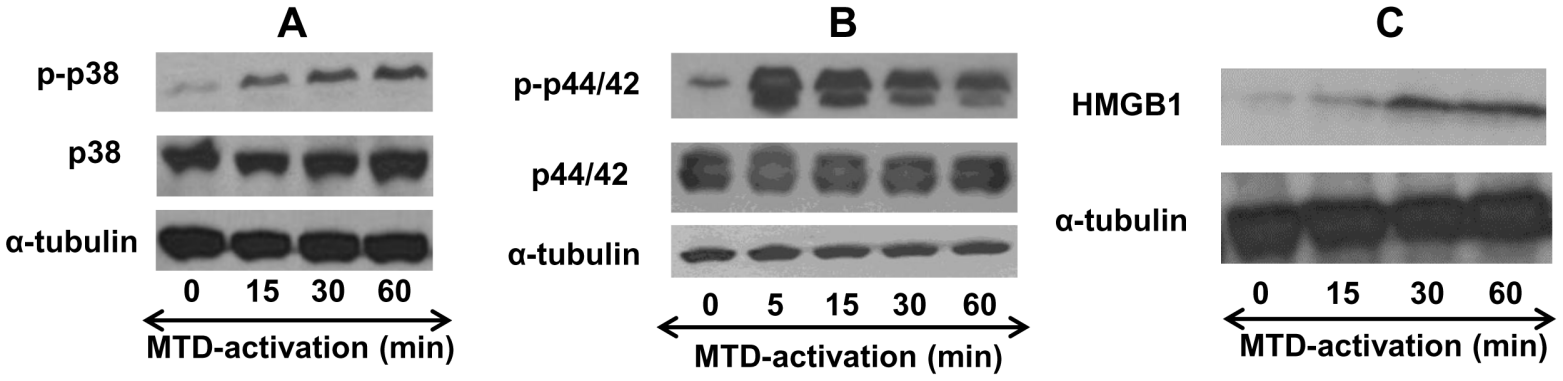
MTD activates MAPK and increases HMGB1 expression in EC. (A) EA cells were seeded on 6-well plates and incubated with MTD (×1/25) for the indicated times. Whole cell lysates were prepared and MAPK (p38) activation was evaluated by Western Blot analysis for phosphorylated p38 (p-p38) and total p38. α-tubulin was used to evaluate loading accuracy. The data are representative of at least three experiments. (B) Similar to 8A, activation of p44/42 MAPK was evaluated by Western Blot analyses for p44/42 and the p-p44/42 species. α-tubulin was used to evaluate loading accuracy. Data are representative of at least three experiments. (C) Similar to 8 A and B, HMGB1 cellular expression was evaluated after MTD stimulation. Data represent the results of at least three different experiments.

Induction of ICAM-1 in endothelial cells is known to be under the control of MAP kinases [17] and we observed here that mtDNA induces ICAM-1 expression (**Figure 6B**). Also, we previously showed that mtDNA only activates p38-MAPK but not p44/42 MAPK (Erk1/2) in PMN whereas MTD activates both kinases [2]. We therefore investigated whether mitochondrial DAMPs could activate endothelial cell MAPKs. EA cells were treated with MTD (×1/40 dilution) for various time periods. Whole cell lysates were then prepared for Western Blot analysis. As seen in **Figure 8A**, MTD caused a rapid phosphorylation of p38-MAPK in EA cells. MTD treatment also resulted in phosphorylation of p44/42 MAPK, although with noticeably different kinetics compared to p38 (**Figure 8B**). These findings are consistent with the conclusion that mitochondrial DAMPs activate endothelial cell MAPK by more than one pathway, much as seen in PMN.

### MTD Up-regulates Endothelial Expression of HMGB1

HMGB1 is a non-histone nuclear protein that can be released directly in response to tissue injury [18] or as a cytokine late in inflammation. HMGB1 is known to activate innate immunity via TLR2, TLR4 and the RAGE receptor [19]. EC exposure to HMGB1 has also been suggested as an important regulator of vascular permeability [20]. We therefore studied whether exposure to MT might result in autocrine or paracrine activation of EC permeability via HMGB1. EA cells were incubated with MTD (×1/40) for different time periods and cell lysates were prepared. Expression levels of HMGB1 protein were evaluated by Western Blot. As shown in **Figure 8C**, expression of HMGB1 by endothelial cells was immediately and strikingly increased by incubation with MTD.

## Discussion

Injury and shock syndromes can induce systemic inflammation and subsequent organ dysfunction. We previously showed that mitochondrial DNA circulates in the plasma at high concentrations after trauma, shock and surgery [1], [2], [21]. Thus tissue destruction leads to the circulation of mitochondrial damage molecules, or DAMPs. We have shown that mitochondrial DAMPs (MTD) are capable of activating inflammation and PMN-mediated lung injury when injected systemically *in vivo* [1], [2]. Moreover, the central role of mitochondrial formyl peptides in PMN activation and function *in vivo* has since been corroborated [22]. The possibility that endothelial cells might participate in the systemic response to MTD released by tissue injury however, has not been studied.

Systemic inflammatory response syndrome (SIRS) and related organ failure syndromes (like ALI/ARDS) are often thought of as reflecting the effects of inflammatory cytokine release on PMN-EC interactions. But cytokine release is the delayed, indirect response to injury seen after intermediate events transduce physical stimuli into immune ‘Danger’ signals. Similarly, release of known endothelial activators like thrombin is strictly limited to sites of wounding. We hypothesized that MTD released from sites of injury might participate in the acute activation PMN-EC interactions that underlie the increased systemic endothelial permeability typical of SIRS and ALI. In this context, we focused our studies on the effects of mitochondrial DAMPs (MTD) on EC activation and permeability in the presence and absence of PMN. Although the concentrations of these molecules under clinical conditions are difficult to ascertain, we have tried to maintain translational relevance by examining the effects of biologic mixtures of mitochondrial DAMPs removed directly from fresh human cells and used at concentrations that produce similar modest activations of PMN [Ca^2+^]_i_ flux. Moreover, we examine the effects of mtDNA at concentrations known to be present in plasma after injury [2]. Although this approach maintains clinical relevance it has drawbacks. Unlike using commercial reagent preparations, individual preparations of MTD or mtDNA is moderately different. Thus application at concentrations with similar bioactivity in one assay does not guarantee the same responses in different assays (*viz.* Figures 1 and 2A, B, C). In fact, MTD consists of a mixture of proteins, lipids and DNA and each preparation likely has a slightly different ratio of these components. Furthermore, it is still unknown which molecules or molecular motifs MTD or mtDNA are responsible for the regulation of endothelial permeability changes. Thus we used mtDNA at concentrations that are observed in trauma patients and whole sonicated mitochondria (MTD) at concentrations that generate Ca^2+^ flux responses associated with chemotaxis to mitochondrial formyl peptides both *in vitro* and in *in vivo* in liver injury models [2], [22].

Using this ‘translational’ approach, we examined the effects of mitochondrial DAMPs on permeability changes in endothelial monolayers and then further evaluated key cell-cell interactions and molecular pathways likely to be associated with the observed endothelial responses. Our findings demonstrate that endothelial cell monolayers (both EA and HPAEC) exposed to MTD show a dose-dependent increase in permeability (**Figure 1** **A&B**). Under these conditions there were no additional changes in permeability when PMN were added (**Figure 1C**). Thus when present in sufficient quantity, MTD can elicit changes in permeability directly, without the need for PMN. This may be of clinical significance where patients are neutropenic or where release of DAMPs is overwhelming. This increase in permeability was associated with increased EC phosphorylation of MAPKs (**Figure 8 A&B**) and [Ca^2+^]_i_ mobilization (**Figures 4C&D** **,** **7A**). These findings show that increased EC permeability after exposure to MTD reflects the activation of cellular inflammatory pathways. The kinetics of p38 and p44/42 activation were also markedly different, suggesting as before [**2**] that MTD activates EC via multiple signal pathways.

MTD dependent permeability increases were prevented by the presence of proteases (**Figure 4**). Thus we show that proteins (and specifically non-formylated proteins) in MTD must play a role in MTD-induced EC permeability changes. Purified mtDNA also affects EC permeability directly. But though mtDNA is present in MTD in high concentration, protein degradation by proteases diminishes MTD’s effect on permeability. This suggests that mitochondrial proteins modify the effects of mtDNA through an additional mechanism. We previously showed that MTD activates PMN through mitochondrial formyl-peptides that act on FPR1 [5]. EC however, clearly do not respond to formyl peptides with either a functional change in permeability (**Figure 5A**) or by change in calcium signaling (**Figure 5B**). This is likely explained by EC lacking FPR1. Interestingly, when MTD was applied to EC, [Ca^2+^]_i_ increased in the absence of extracellular calcium implicating release of endoplasmic Ca^2+^ stores. Also, the addition of calcium to the medium did not trigger calcium influx (**Figure 4 C&D**). Moreover, the morphology of the calcium increase was not typical of a G-protein coupled receptor-induced store depletion. Rather, the increases resembled a response to the membrane-permeable diacylglycerol analog 1-oleoyl-2-acetyl-*sn*-glycerol (OAG) [23]. Since protease treatments completely abolish calcium mobilization it is unlikely that a mitochondrial lipid is involved. Thus further investigation will be needed to delineate the mechanisms by which MTD-induced Ca^2+^ mobilization occurs in EC. The data presented here however, clearly show that the FPR-1 and 2-like receptors are likely not involved.

Unlike exposure to MTD (**Figure 1A**), exposure to purified mtDNA caused only short-lived spikes in EC permeability (**Figure 2A**). But when EC were exposed to mtDNA in the presence of PMN, prolonged responses similar to those seen after exposure to MTD were found (**Figure 2A**). Thus mtDNA can increase EC permeability directly, or indirectly through PMN-EC paracrine crosstalk. Externally applied mtDNA localized to endosomes as confirmed by confocal microscopy (**Figure 3**) and activated EC via mechanisms that were inhibitable by CQ (an inhibitor of endosomal acidification) or ODNs that target endosomal TLR receptors for CpG DNA (**Figure 2 B&C**). It is still not clear which TLR(s) mtDNA binds to in EC endosomes, although recent work shows that the effects of mtDNA on cardiac muscle are at least partially due to its binding to TLR9 [24].

PMN adherence to EC commonly precedes increased permeability and adding PMN to EC monolayers increased mtDNA-induced permeability. We therefore examined whether mtDNA increased PMN adherence to EC. mtDNA increased PMN adherence to EC much like exposure to E.coli DNA or LPS (**Figure 6A**). We examined the underlying mechanisms and found that exposure to mtDNA and the associated increase in adherence were associated with dramatically increased expression of the adhesion molecules ICAM-1 and E-selectin in EC and their counter-ligands CD18 and L-selectin in PMN (**Figure 6 B, C, and D**). These findings support the concept that MTD mobilization by direct cellular trauma can participate in pathologic PMN-EC interactions.

Elevations of cytosolic calcium ([Ca^2+^]_i_) are a common mechanism of immune cell activation. We found mtDNA-treated PMN to have an increased Ca^2+^ influx response to TG (**Figure 7A**). Increased Ca^2+^ influx is a critical event in trauma and traumatic ALI/ARDS [9], [11], [15]. Since PMN Ca^2+^ influx depends on S1P synthesis by Sphk1 [16], we next examined whether exposure to mtDNA increased PMN Sphk1 expression. As shown in **Figure 7B**, Sphk1 expression in PMN (at least at the mRNA level) was increased by mtDNA, suggesting that mtDNA activates PMN by increasing Sphk1 and S1P, thus leading to enhanced store-operated calcium entry. S1P expressed intracellularly can act as a “calcium influx factor” and increase calcium influx. We believe such S1P synthesis may contribute to PMN activation and subsequent increases in endothelial permeability, but further investigations will be needed to test this hypothesis.

Such further investigations will involve direct evaluation of intracellular S1P levels as we have shown previously [16] and identification of the channels mediating SOCE and increased Ca^2+^ influx after mtDNA exposure in this system. It is widely known that extracellular S1P acts on the EC plasma membrane receptors (S1PR_1–5_) to enhance barrier integrity [25], [26]. But there is also strong evidence that ***intracellular*** S1P synthesis activates store-operated Ca^2+^ channels. Increases in [Ca^2+^]_i_ due to SOCE in PMN or EC could then activate pathways leading to increased vascular permeability. Studies by our lab and others have suggested that S1P acts as a second messenger and has an important non-receptor mediated role in the regulation of [Ca^2+^]_i_ mobilization and cell permeability [11], [16], [27]–[29] after exposure to GPCR ligands like thrombin, PAF or ATP, or to endoplasmic-reticular Ca^2+^ store depletion by other signaling agents [16]. Thus we think understanding Sphk1 activation may be critical in understanding endothelial permeability.

The increase in Sphk1 induced by mtDNA treatment (**Figure 7B**) is therefore a novel and potentially important finding. Our studies are still in progress, but the data suggest mitochondrial DAMPs may activate Sphk1, S1P production and regulate calcium influx that modulates endothelial permeability. All of the formylated proteins in MTD are capable of activating PMN [30] and many others may activate EC via SOCE mechanisms. The protection of EC from increased permeability by protease pre-treatment of the MTD shows that MT proteins are indeed important. Moreover, diversity in EC Ca^2+^ flux responses may be an important source of the diversity in endothelial responses seen in different vascular beds.

Finally, we show here for the first time that endothelial cell expression of HMGB1 is up-regulated in inflammatory environments and that sterile mitochondrial motifs can activate HMGB1 expression. Although HMGB1 has been thought to act as a late cytokine mediator of sepsis that activates cells via innate immune receptors [20], [31], we now know that HMGB1 can also be released early after injury as a damage marker [18]. Here, rapid induction of HMGB1 might create an autocrine or paracrine environment conducive to increased adhesion and endothelial permeability. Interestingly, the induction of HMGB1 in EC suggests a mechanism by which EC could induce PMN inflammation as opposed to PMN activating EC. Investigations of HMGB1 as a potential ‘short-arc’ inducer of local autocrine/paracrine inflammatory responses are ongoing and will be the subject of later reports.

The ability of DAMPs derived from mitochondria to initiate changes in endothelial permeability has not been previously studied. The events described here are actually responses to a spectrum of molecular species that can act on both EC and PMN. The current work establishes that release of molecular motifs from the mitochondria of disrupted, dead or dying cells may be a critical event in determining early systemic endothelial response to injury. Subsequent studies will be aimed at determining the dominant active agonists and the pathways by which they act.
